# Vesicular Stomatitis Virus-Based Ebola Vaccine Is Well-Tolerated and Protects Immunocompromised Nonhuman Primates

**DOI:** 10.1371/journal.ppat.1000225

**Published:** 2008-11-28

**Authors:** Thomas W. Geisbert, Kathleen M. Daddario-DiCaprio, Mark G. Lewis, Joan B. Geisbert, Allen Grolla, Anders Leung, Jason Paragas, Lennox Matthias, Mark A. Smith, Steven M. Jones, Lisa E. Hensley, Heinz Feldmann, Peter B. Jahrling

**Affiliations:** 1 National Emerging Infectious Diseases Laboratories Institute, Boston, Massachusetts, United States of America; 2 Department of Microbiology, Boston University School of Medicine, Boston, Massachusetts, United States of America; 3 Department of Medicine, Boston University School of Medicine, Boston, Massachusetts, United States of America; 4 Department of Pathology, Uniformed Services University of the Health Sciences, Bethesda, Maryland, United States of America; 5 Integrated Research Facility at Fort Detrick, National Institute of Allergy and Infectious Diseases, National Institutes of Health, Bethesda, Maryland, United States of America; 6 Virology Division, BIOQUAL, Rockville, Maryland, United States of America; 7 BIOQUAL, Rockville, Maryland, United States of America; 8 Special Pathogens Program, National Microbiology Laboratory, Public Health Agency of Canada, Winnipeg, Manitoba, Canada; 9 Emerging Viral Pathogens Section, National Institute of Allergy and Infectious Diseases, National Institutes of Health, Bethesda, Maryland, United States of America; 10 Pathology Division, U.S. Army Medical Research Institute of Infectious Diseases, Fort Detrick, Maryland, United States of America; 11 Department of Immunology, University of Manitoba, Winnipeg, Manitoba, Canada; 12 Medical Microbiology, University of Manitoba, Winnipeg, Manitoba, Canada; University of Wisconsin-Madison, United States of America

## Abstract

Ebola virus (EBOV) is a significant human pathogen that presents a public health concern as an emerging/re-emerging virus and as a potential biological weapon. Substantial progress has been made over the last decade in developing candidate preventive vaccines that can protect nonhuman primates against EBOV. Among these prospects, a vaccine based on recombinant vesicular stomatitis virus (VSV) is particularly robust, as it can also confer protection when administered as a postexposure treatment. A concern that has been raised regarding the replication-competent VSV vectors that express EBOV glycoproteins is how these vectors would be tolerated by individuals with altered or compromised immune systems such as patients infected with HIV. This is especially important as all EBOV outbreaks to date have occurred in areas of Central and Western Africa with high HIV incidence rates in the population. In order to address this concern, we evaluated the safety of the recombinant VSV vector expressing the *Zaire ebolavirus* glycoprotein (VSVΔG/ZEBOVGP) in six rhesus macaques infected with simian-human immunodeficiency virus (SHIV). All six animals showed no evidence of illness associated with the VSVΔG/ZEBOVGP vaccine, suggesting that this vaccine may be safe in immunocompromised populations. While one goal of the study was to evaluate the safety of the candidate vaccine platform, it was also of interest to determine if altered immune status would affect vaccine efficacy. The vaccine protected 4 of 6 SHIV-infected macaques from death following ZEBOV challenge. Evaluation of CD4+ T cells in all animals showed that the animals that succumbed to lethal ZEBOV challenge had the lowest CD4+ counts, suggesting that CD4+ T cells may play a role in mediating protection against ZEBOV.

## Author Summary

Ebola virus is among the most lethal microbes known to man, with case fatality rates often exceeding 80%. Since its discovery in 1976, outbreaks have been sporadic and geographically restricted, primarily to areas of Central Africa. However, concern about the natural or unnatural introduction of Ebola outside of the endemic areas has dramatically increased both research interest and public awareness. A number of candidate vaccines have been developed to combat Ebola virus, and these vaccines have shown varying degrees of success in nonhuman primate models. Safety is a significant concern for any vaccine and in particular for vaccines that replicate in the host. Here, we evaluated the safety of our replication-competent vesicular stomatitus virus (VSV)-based Ebola vaccine in SHIV-infected rhesus monkeys. We found that the vaccine caused no evidence of overt illness in any of these immunocompromised animals. We also demonstrated that this vaccine partially protected the SHIV-infected monkeys against a lethal Ebola challenge and that there appears to be an association with levels of CD4+ lymphocytes and survival. Our study suggests that the VSV-based Ebola vaccine will be safe in immunocompromised populations and supports further study and development of this promising vaccine platform for its use in humans.

## Introduction

Ebola virus (EBOV) has been associated with sporadic episodes of hemorrhagic fever (HF) that produce severe disease in infected patients. Mortality rates in outbreaks have ranged from 50% for *Sudan ebolavirus* (SEBOV) to up to 90% for *Zaire ebolavirus* (ZEBOV) (reviewed in [1]). A recent outbreak caused by an apparently new species of EBOV in Uganda appears to be less pathogenic than SEBOV or ZEBOV with a preliminary case fatality rate of about 25% [2]. EBOV is also considered to have potential as a biological weapon and is categorized as a Category A bioterrorism agent by the Centers for Disease Control and Prevention [3]–[5].

While there are no vaccines or postexposure treatment modalities available for preventing or managing EBOV infections there are at least four different vaccine systems that have shown promise in completely protecting nonhuman primates against a lethal EBOV challenge [6]–[12]. Of these prospective EBOV vaccines two systems, one based on a replication-defective adenovirus and the other based on a replication-competent vesicular stomatitis virus (VSV), were shown to provide complete protection when administered as a single injection vaccine [7]–[9]. Most intriguingly, the VSV-based vaccine is the only vaccine which has shown any utility when administered as a postexposure treatment [13],[14].

Of these two leading EBOV vaccine candidates that can confer protection as single injection vaccines each has advantages and disadvantages. Adenovirus vectors are highly immunogenic as documented by clinical trials evaluating gene transfer efficacy and immune responses. Because they are replication-defective adenovirus vectors are also perceived to be safer for human use than a replication-competent vaccine. The most significant challenge for the adenovirus-based vaccines is the concern that a significant portion of the global population has pre-existing antibodies against the adenovirus vector which may affect efficacy [15]–[17] and has performed poorly as a vaccine vector in recent clinical trials [18]–[19]. In contrast, pre-existing immunity against VSV in human populations is negligible [20] and efficacy is likely greater with replication-competent vectors. The main concern with the VSV vaccine vector is that replication-competent vectors may present more significant safety challenges in humans particularly those with altered immune status.

Because EBOV outbreaks in man have occurred exclusively in Central and Western Africa, the populations in this region are among those that may benefit from the development and availability of an EBOV vaccine. However, populations in this region are among the most medically disadvantaged in the world. In particular, the prevalence of individuals with a compromised immune system is high and HIV infections rates range up to 10% or more in this area [21]. While the VSV vaccine vector has been enormously successful in protecting healthy immunocompetent animals against EBOV [7],[13],[14], we are uncertain as to how these vectors would behave in individuals with altered or compromised immune systems. Therefore, we conducted a study to assess the pathogenicity and protective efficacy of the recombinant VSV-based ZEBOV vaccine vector in rhesus macaques that were infected with simian-human immunodeficiency virus (SHIV) which is known to deplete the populations of naive CD4+ T cells, naive CD8+ T cells, and memory CD4+ T cells in these animals [22],[23]. In order to take into account the degree or severity of compromised immune function animals were selected with varying degrees of CD4+ T cell loss.

## Methods

### Vaccine Vectors and Challenge Virus

The recombinant VSV expressing the glycoprotein (GP) of ZEBOV (strain Mayinga) (VSVΔG/ZEBOVGP) was generated as described recently using the infectious clone for the VSV, Indiana serotype [24]. ZEBOV (strain Kikwit) was isolated from a patient of the ZEBOV outbreak in Kikwit in 1995 [25].

### Animal Studies

Nine filovirus-seronegative adult rhesus macaques (*Macaca mulatta*) (5–10 kg) were used for these studies. The macaques were infected three months prior to the current study with SHIV162p3 (kindly provided by Dr. Ranajit Pal, Advanced BioScience Laboratories, Inc., Kensington, MD). These animals all had clinical laboratory evidence of SHIV infection as evidenced by reduced CD4+ T cell counts, decreased ratios of CD4+/CD8+ T cells (Table 1) and the presence of SHIV in plasma of four out of nine animals (Table 2). Six of the nine SHIV-infected animals were vaccinated by i.m. injection with ∼1×10∧7 recombinant VSVΔG/ZEBOVGP. Three animals served as placebo controls and were injected in parallel with saline. All six VSVΔG/ZEBOVGP-vaccinated animals and two of the three control animals were challenged 31 days after the single dose immunization with 1000 pfu of ZEBOV (strain Kikwit). The monkeys were challenged with the heterologous Kikwit strain of ZEBOV as our macaque models have been developed and characterized using this strain [1],[26]. Animals were closely monitored for evidence of clinical illness (e.g., temperature, weight loss, changes in complete blood count, and blood chemistry) during both the vaccination and ZEBOV challenge portions of the study. In addition, VSVΔG/ZEBOV and ZEBOV viremia and shedding were analyzed after vaccination and challenge, respectively. Animals were given physical exams and blood and swabs (nasal, oral, rectal) were collected at 2, 4, 7, 10, 14, 21, 28, and 31 days after vaccination and on days 3, 6, 10, 15, and 28 after ZEBOV challenge. The vaccination portion of the study was conducted at BIOQUAL and was approved by NIAID, BIOQUAL, and USAMRIID Laboratory Animal Care and Use Committees. The ZEBOV challenge was performed in BSL-4 biocontainment at USAMRIID and was approved by the USAMRIID Laboratory Animal Use Committee. Animal research was conducted in compliance with the Animal Welfare Act and other Federal statues and regulations relating to animals and experiments involving animals and adheres to the principles stated in the *Guide for the Care and Use of Laboratory Animals*, National Research Council, 1996. Both facilities used are fully accredited by the Association for Assessment and Accreditation of Laboratory Animal Care International.

**Table 1:** Pre-vaccination hematology of SHIV-infected rhesus macaques.

Animal No.	Pre-SHIV CD4	Post-SHIV CD4	CD4 % Drop	Pre-SHIV CD4/CD8	Post-SHIV CD4/CD8	CD4/CD8 %Change
Subject 1	2610	541	79	1.17	0.45	61
Subject 2	1207	627	48	1.03	0.44	57
Subject 3	861	595	31	0.68	0.48	29
Subject 4	1380	681	51	0.64	0.26	59
Subject 5	509	83	84	0.86	0.09	89
Subject 6	1193	42	96	0.92	0.03	97
Control 1	846	329	61	1.16	0.16	86
Control 2	731	289	60	1.00	0.29	71
Control 3	651	288	56	0.59	0.25	58

**Table 2:** SHIV load determined by a nucleic acid sequence-based amplification assay.

Animal No.	Day 0*	Day 2	Day 7	Day 10	Day 28
Subject 1	<40	<40	<40	<40	<40
Subject 2	<40	<40	<40	<40	<40
Subject 3	<40	<40	400	1920	<40
Subject 4	<40	<40	<40	<40	<40
Subject 5	3420	7760	9700	8720	22480
Subject 6	22760	9900	11800	21880	8380
Control 1	900	400	960	1780	<40
Control 2	<40	<40	<40	<40	<40
Control 3	<40	<40	<40	<40	<40

*: Days after vaccination; values listed as genomes/ml of blood.

### Hematology and Serum Biochemistry

Total white blood cell counts, white blood cell differentials, red blood cell counts, platelet counts, hematocrit values, total hemoglobin, mean cell volume, mean corpuscular volume, and mean corpuscular hemoglobin concentration were determined from blood samples collected in tubes containing EDTA, by using a laser-based hematologic Analyzer (Coulter Electronics, Hialeah, FL, USA). The white blood cell differentials were performed manually on Wright-stained blood smears. Serum samples were tested for concentrations of albumin (ALB), amylase (AMY), alanine aminotransferase (ALT), aspartate aminotransferase (AST), alkaline phosphatase (ALP), gamma-glutamyltransferase (GGT), glucose (GLU), cholesterol (CHOL), total protein (TP), total bilirubin (TBIL), blood urea nitrogen (BUN), and creatinine (CRE) by using a Piccolo Point-Of-Care Blood Analyzer (Abaxis, Sunnyvale, CA, USA).

### Flow Cytometry for Circulating Cell Populations

100 ul of whole blood was added to a 12×75 tube and incubate with the antibodies for 15 minutes at room temperature. The samples was then lysed and fixed in 1% paraformaldehyde and washed three times in PBS. Samples were analyzed on a Becton Dickinson FACS Calibur (Becton Dickinson, San Jose, CA). All antibodies were purchased from Becton Dickinson; clones used were CD3 – SP34, CD4 – L200, CD8 – RPA-T8 and CD20 – 2H7.

### Detection of SHIV

For measurement of plasma SIV RNA levels, a quantitative TaqMan RNA reverse transcription-PCR (RT-PCR) assay (Applied Biosystems, Foster City, CA) was used, which targets a conserved region of SIV gag and has an accurate detection limit as low as 200 RNA copies/ml. Briefly, isolated plasma viral RNA was used to generate cDNA using One-Step RT-PCR Master Mix (Applied Biosystems). The samples were then amplified as previously described [27] with the following PCR primer/probes: SIV-F 5′ AGTATGGGCAGCAAATGAAT 3′ (forward primer), SIV-R 5′TTC TCTTCTGCGTG AATGC 3′ (reverse primer), SIV-P 6FAM-AGATTTGGATTAGCAGAAAGCCTGTTG GA-TAMRA (TaqMan probe) in a 7700 Sequence Detection System (40 cycles of 95°C, 15 seconds, and 60°C, 1 minute). The signal was then compared to a standard curve of known concentrations to determine the viral copies present in each sample. The assay lower limit was 40 copies/ml.

### Detection of VSV and ZEBOV

RNA was isolated from blood and swabs using Tripure Reagent (INVITROGEN, Grand Island, New York). For the detection of VSV we used a Q-RT-PCR assay targeting the matrix gene (nt position 2497–2556, AM690337). ZEBOV RNA was detected using a Q-RT-PCR assay targeting the L gene (nt position 13874–13933, AY354458). The low detection limit for this ZEBOV assay is 0.1 pfu/ml of plasma. Virus titration was performed by plaque assay on Vero E6 cells from all blood and selected organ (liver, spleen, lung, kidney, adrenal gland, pancreas, axillary lymph node, inguinal lymph node, mesenteric lymph node, ovary or testis, and brain) and swab samples. Briefly, increasing 10-fold dilutions of the samples were adsorbed to Vero E6 monolayers in duplicate wells (0.2 ml per well); thus, the limit for detection was 25 pfu/ml.

### Humoral Immune Response

IgG antibodies against ZEBOV were detected with an Enzyme-Linked Immunosorbent Assay (ELISA) using purified virus particles as an antigen source as previously described [7],[9].

### Histology and Immuohistochemistry

Necropsies were performed on each animal and selected tissues were collected for histological analysis. Histology and immunohistochemistry were performed as previously described for ZEBOV-infected monkeys [26].

## Results

**Figure 1:**
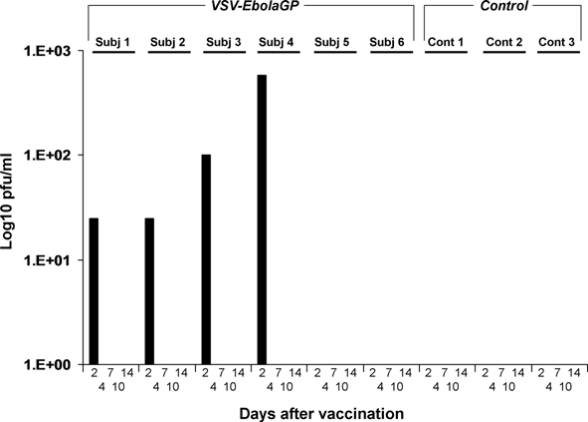
Plasma levels of VSVΔG/ZEBOVGP from rhesus macaques after vaccination.

We employed nine SHIV-infected rhesus macaques, of which six animals were vaccinated by i.m. injection with a single dose of VSVΔG/ZEBOVGP (Subjects #1–6) and the remaining three animals (Controls #1–3) received sterile saline. The animals were monitored closely for clinical symptoms and shedding of recombinant VSVs. None of the animals vaccinated with VSVΔG/ZEBOVGP or treated with saline showed overt fever or any evidence of clinical illness during the 31 day vaccination period. Importantly, no evidence of reaction at the vaccine injection site was noted among any of the VSVΔG/ZEBOVGP-vaccinated animals nor was any change noted in activity or behavior during the vaccination phase of the study (day 0 to day 31 after vaccination). In addition, no changes were detected in hematology or clinical chemistry following vaccination. A mild VSVΔG/ZEBOVGP viremia (<10^3^ pfu/ml) was detected only on day 2 after vaccination by virus isolation (Figure 1) and RT-PCR (data not shown) in four of the six VSVΔG/ZEBOVGP-immunized macaques (Subjects #1, 2, 3, 4). Surprisingly, the two animals with the lowest CD4+ counts (subjects #5, 6) never showed any detectable level of VSV viremia. VSVΔG/ZEBOVGP was undetectable in all analyzed swab samples (data not shown). Thus, vaccination led to a transient viremia from virus replication at as yet undetermined sites but no virus shedding of the vaccine virus.

**Figure 2:**
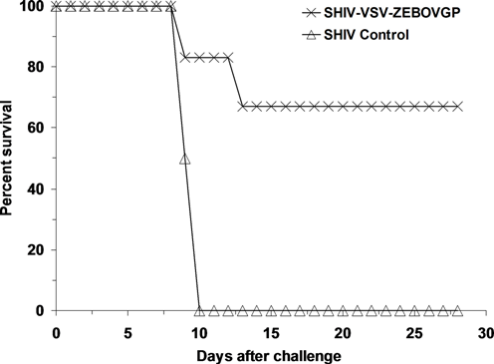
Kaplan-Meier survival curves for SHIV-infected rhesus macaques vaccinated with VSVΔG/ZEBOVGP and challenged 31 days later with ZEBOV.

Following successful completion of the safety portion of the study all six of the VSVΔG/ZEBOVGP-vaccinated SHIV-infected monkeys and two of the three placebo control SHIV-infected monkeys were challenged 31 days after the single immunization by i.m. injection with 1000 pfu of ZEBOV (strain Kikwit). Four of the six VSVΔG/ZEBOVGP-vaccinated SHIV-infected monkeys and both of the placebo control animals started to show clinical signs of disease on day 6 after challenge including fever (Subject # 1, 2 and Control #1, 2) and lymphopenia and thrombocytopenia (Subject #2, 5, 6 and Control #1, 2) (Table 3). Disease progressed in two of the VSVΔG/ZEBOVGP-vaccinated SHIV-infected monkeys (Subject #5 and 6) and both of the placebo control animals with the development of additional evidence of clinical illness including increased levels of serum enzymes associated with liver function, depression, anorexia, and the appearance of macular rashes (Table 3). All four of these animals succumbed to the ZEBOV challenge with the two VSVΔG/ZEBOVGP-vaccinated monkeys expiring on days 9 (Subject #6) and 13 (Subject #5) and the placebo controls succumbing on days 9 (Control #1) and 10 (Control #2) after ZEBOV challenge (Figure 2). Disease did not progress in the two VSVΔG/ZEBOVGP-vaccinated SHIV-infected monkeys that were febrile (Subjects #1, 2) and had changes in hematology values on day 6 (Subjects #2) and both of these animals remained healthy and survived the ZEBOV challenge (Figure 2). The remaining VSVΔG/ZEBOVGP-vaccinated macaques (Subject #3, 4) never showed any evidence of clinical illness and survived (Figure 2). Interestingly, the VSVΔG/ZEBOVGP-vaccinated macaques that succumbed were the two animals with the most significant reduction in CD4+ T cells (84%, 96%) (Table 1), the lowest total CD4+ T cell counts (83, 42) (Table 1), the highest SHIV viremia (Table 2), and no evidence for VSV viremia (Figure 1) suggesting that CD4+ T cells may play a role in protection.

**Table 3:** Clinical findings in SHIV-infected rhesus monkeys challenged with ZEBOV.

Animal	Day 0–8	Day 9–10	Day 11–28	Day of Death
Subject 1	Fever (6)			Survived
Subject 2	Fever (6), Lymphopenia (6), Thrombocytopenia (6)	Thrombocytopenia (10)		Survived
Subject 3				Survived
Subject 4				Survived
Subject 5	Lymphopenia (6), Thrombocytopenia (6)	Anorexia (10), Depression (10), ALT↑ (10), AST↑↑↑ (10), BUN↑ (10), GGT↑ (10)	Anorexia (11–13), Depression (11–13), Moderate rash (12–13), ALB↓ (13), ALP↑↑ (13), ALT↑↑↑ (13), AST↑↑↑ (13), AMY↓ (13), BUN↑↑↑ (13), CRE↑ (13), GGT↑↑↑ (13), UA↑ (13), GLU↓ (13)	Day 13
Subject 6	Anorexia (7–8), Depression (7–8), Mild rash (8), Lymphopenia (6), Thrombocytopenia (6)			Day 9
Control 1	Fever (6), Anorexia (7–8), Depression (8), Mild rash (6–8), Lymphopenia (6), Thrombocytopenia, ALP↑ (6), AST↑↑↑ (6)	Anorexia (9), Depression (9), Moderate rash (9), Epistaxis (9), Thrombocytopenia (9), ALP↑↑↑ (9), ALT↑↑↑ (9), AST↑↑↑ (9), BUN↑↑↑ (9), CRE↑↑↑ (9), GGT↑↑↑ (9), GLU↓ (9)		Day 9
Control 2	Fever (6), Anorexia (8), Depression (8), Lymphopenia (6), Thrombocytopenia (6), ALP↑↑ (6), AST↑ (6)	Anorexia (9), Depression (9), Moderate rash (9)		Day 10

Fever is defined as a temperature more than 2.5 °F over baseline or at least 1.5 °F over baseline and ≥103.5 °F.

Mild rash: focal areas of petechiae covering less than 10% of the skin; moderate rash: areas of petechiae covering between 10% and 40% of the skin; severe rash: areas of petechiae and/or echymosis covering more than 40% of the skin.

Lymphopenia and thrombocytopenia defined by ≥35% drop in numbers of lymphocytes and platelets, respectively.

Alkaline phosphatase (ALP), alanine aminotransferase (ALT), aspartate aminotransferase (AST), gamma-glutamyltransferase (GGT), blood urea nitrogen (BUN), creatinine (CRE), uric acid (UA), Albumin (ALB), Amylase (AMY), Glucose (GLU).

↑ = 2–3 fold increase; ↑↑ = 4–5 fold increase; ↑↑↑ = >5 fold increase; ↓ = 2–3 fold decrease.

( ) Days after ZEBOV challenge are shown in parentheses.

Blood samples were analyzed after challenge for evidence of ZEBOV replication by plaque assay and RT-PCR. By day 6, both of the placebo control animals developed high ZEBOV titers in plasma as detected by plaque assay (>10^4.5^ log pfu/ml) (Table 4). In comparison, only one of the VSVΔG/ZEBOVGP-vaccinated monkeys (Subject #6) showed a ZEBOV viremia at day 6 by plaque assay (∼10^2^ log pfu/ml) (Table 4). ZEBOV was detected in a second VSVΔG/ZEBOVGP-vaccinated monkey (Subject #5) by day 10 (∼10^4.2^ log pfu/ml). RT-PCR was more sensitive and showed evidence of ZEBOV in plasma of this animal (Subject #5) at day 6. In addition, RT-PCR was more sensitive in detecting ZEBOV in swabs which were positive on a number of samples derived from Subject #5 at day 6 and day 10 (Table 4). In contrast, no ZEBOV was detected in the plasma by virus isolation or RT-PCR in the four VSVΔG/ZEBOVGP-vaccinated monkeys that survived ZEBOV challenge. Moreover, no evidence for reactivation of VSVΔG/ZEBOVGP was detected from any blood or swab sample from any animal after ZEBOV challenge (data not shown). Although we failed to detect ZEBOV viremia in the two surviving animals that were clinically ill (Subject #1 and 2) at days 3, 6, 10, and 14 after ZEBOV challenge we cannot exclude the possibility that these animals had low levels of circulating ZEBOV at time points not evaluated.

**Table 4:** Viral load in SHIV-infected rhesus monkeys after ZEBOV challenge.

Animal No.	Plasma		PBMC		Throat		Nasal		Rectal		Vaginal	
	D 6	D 10	D 6	D 10	D 6	D 10	D 6	D 10	D 6	D 10	D 6	D 10
Subject 1	0*	0	NT	NT	0	0	0	0	NT	NT	NT	NT
	(−)	(−)	(−)	(−)	(−)	(−)	(−)	(−)	(−)	(−)	(−)	(−)
Subject 2	0	0	NT	NT	0	0	0	0	NT	NT	NT	NT
	(−)	(−)	(−)	(−)	(−)	(−)	(−)	(−)	(−)	(−)	(−)	(−)
Subject 3	0	0	NT	NT	0	0	0	0	NT	NT	NA	NA
	(−)	(−)	(−)	(−)	(−)	(−)	(−)	(−)	(−)	(−)	NA	NA
Subject 4	0	0	NT	NT	0	0	0	0	NT	NT	NA	NA
	(−)	(−)	(−)	(−)	(−)	(−)	(−)	(−)	(−)	(−)	NA	NA
Subject 5	0	4.2	NT	NT	0	2.2	0	0	NT	NT	NT	NT
	(+)	(+)	(+)	(−)	(−)	(+)	(−)	(+)	(−)	(+)	(−)	(+)
Subject 6	2.0		NT		0		0		NT		NT	
	(+)		(+)		(−)		(−)		(−)		(−)	
Control 1	5.4		NT		0		0		NT		NT	
	(+)		(+)									
Control 2	4.9		NT		0		0		NT		NT	
	(+)		(+)		(−)		(−)		(−)		(−)	

*, Log 10 pfu of ZEBOV per ml of plasma; (+), sample positive for ZEBOV by RT-PCR; (−), sample negative for ZEBOV by RT-PCR; NT, not tested; NA, not applicable.

The four surviving VSVΔG/ZEBOVGP-vaccinated macaques (Subjects #1, 2, 3, 4) were euthanized 28 days after the ZEBOV challenge to perform a virological and pathological examination of tissues. Organ infectivity titration from these four animals showed no evidence of ZEBOV in any of the tissues examined. In comparison, ZEBOV was recovered from tissues of both VSVΔG/ZEBOVGP-vaccinated animals that succumbed (Subject #5, 6) and both SHIV-infected control animals. Organ titers of infectious ZEBOV were consistent with values previously reported for immunocompetent ZEBOV-infected rhesus macaques [27],[28]. VSVΔG/ZEBOVGP was not recovered in any of the tissues examined from any animal on this study.

**Figure 3:**
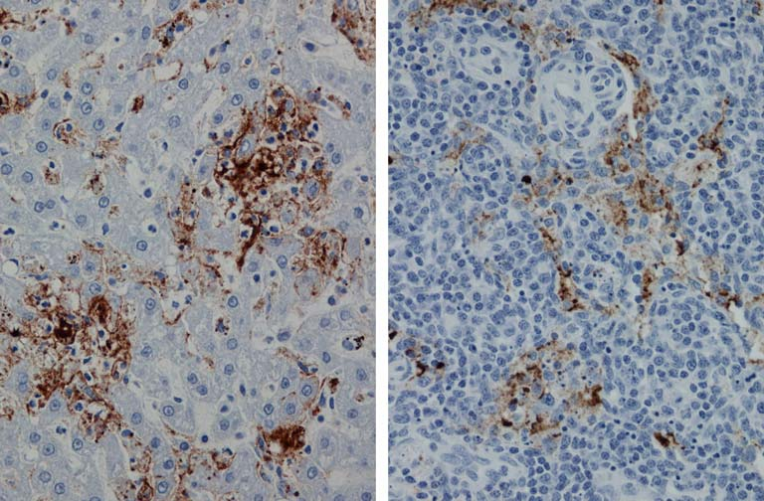
Tissues from SHIV-infected rhesus monkeys vaccinated with VSVΔG/ZEBOVGP and challenged 31 days later with ZEBOV. (Left panel) Immunohistochemical staining of liver from animal that succumbed on day 9 (Subject 6) for ZEBOV. Note abundance of EBOV antigen (brown) associated with sinusoids. (Right panel) Immunohistochemical staining of lymph node from animal that succumbed on day 13 (Subject 5) for ZEBOV. Note localization of ZEBOV antigen (brown) associated with macrophages and dendritiform cells. Original magnifications, ×20.

Pathological and immunohistochemical evaluation of tissues from the four VSVΔG/ZEBOVGP-vaccinated animals (Subjects #1, 2, 3, 4) that survived ZEBOV challenge showed no evidence of ZEBOV antigen. In contrast, ZEBOV antigen was readily detected in typical target organs (e.g., liver, spleen, adrenal gland, lymph nodes) of the two VSVΔG/ZEBOVGP-vaccinated animals that succumbed to ZEBOV challenge (Subject #5, 6) (Figure 3) and the two placebo controls. Lesions and distribution of ZEBOV antigen in these macaques was consistent with results reported in other studies [27],[29].

**Figure 4:**
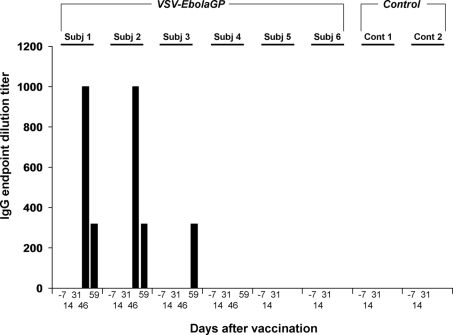
Circulating levels of IgG against ZEBOV from SHIV-infected rhesus macaques vaccinated with VSVΔG/ZEBOVGP and challenged 31 days later with ZEBOV.

While cellular immune responses against ZEBOV GP in macaques vaccinated with VSVΔG/ZEBOVGP vectors have been difficult to detect before challenge in previous studies [7], humoral immune responses have been more robust and consistent ([7]; TW Geisbert, unpublished observations). Therefore, we measured the antibody responses of the rhesus macaques vaccinated with VSVΔG/ZEBOVGP before vaccination (day −7), after vaccination (day 14 and day 31), and after ZEBOV challenge (day 46 and day 59 after vaccination) by IgG ELISA. None of the six VSVΔG/ZEBOVGP-vaccinated macaques developed IgG antibody titers against the ZEBOV GP by the day of ZEBOV challenge (Figure 4). Two animals (Subjects #1, 2) developed modest IgG antibody titers against ZEBOV by day 15 after ZEBOV challenge (day 46 after vaccination) while a third animal developed a titer by day 28 after ZEBOV challenge (day 59 after vaccination) (Figure 4).

## Discussion

An often raised concern regarding the use of the recombinant VSV vaccine platform in humans is related to the fact that this is a replication-competent vaccine, and thus demonstration of safety is of paramount importance. Taking into account our previous work it is not surprising that the VSVΔG/ZEBOVGP was tolerated well in our SHIV-infected macaques. Specifically, we failed to observe evidence of any adverse events in a large cohort of over 90 macaques receiving VSV vectors expressing different GPs from viral HF agents (38 cynomolgus macaques and 3 rhesus macaques vaccinated with VSVΔG/ZEBOVGP; 12 cynomolgus macaques and 3 rhesus macaques vaccinated with VSV expressing SEBOV GP; 29 cynomolgus macaques and 3 rhesus macaques vaccinated with VSV expressing the Marburg virus GP; and 6 cynomolgus macaques vaccinated with VSV expressing the Lassa GP) ([7],[30],[31]; TW Geisbert, H Feldmann, and SM Jones unpublished observations). We have also failed to observe any adverse events in a variety of immunocompetent laboratory mice (different inbred strains), outbred guinea pigs (Hartley strain) and goats vaccinated with the above mentioned VSV vectors at doses ranging from 2×10^0^–2×10^5^ pfu ([24],[32]; SM Jones and H Feldmann, unpublished observations). More recently we have also demonstrated that vaccination of severely immunocompromised SCID mice with 2×10^5^ pfu of the VSV-based ZEBOV vaccine (VSVΔG/ZEBOVGP) resulted in no clinical symptoms [32]. While transient VSV viremia in this study was only observed in surviving macaques but not in animals that had succumbed to ZEBOV challenge (Figure 1), viremia data from previous studies [7],[30],[31] do not support any correlation between VSV viremia and survival. In addition, no evidence for vaccine vector shedding was detected in this study supporting previous results [7],[30],[31] with no compelling evidence to suggest that occasional virus shedding (only detected by RT-PCR; negative on virus isolation) would lead to vaccine vector transmission.

The VSV glycoprotein exchange vector that we employed in this study has also shown promise as a preventive vaccine and postexposure treatment against Marburg HF [30],[33] and as a preventive vaccine against Lassa fever in nonhuman primates [31]. Similar recombinant VSV vectors have been evaluated in animal models as vaccine candidates for a number of viruses that cause disease in humans including HIV-1, influenza virus, respiratory syncytial virus, measles virus, herpes simplex virus type 2, hepatitis C virus, and severe acute respiratory syndrome coronavirus [34]–[40]. Many of these studies have employed VSV vectors that maintained either the entire VSV glycoprotein (G) or the transmembrane and/or cytoplamic domains of this protein to facilitate more efficient incorporation of the foreign antigen. It is known that VSV G is an important VSV protein associated with pathogenicity [38],[41]. It has been shown that truncation of the cytoplasmic tail has greatly reduced vector pathogenicity in mice following intranasal inoculation indicating the importance of this domain for pathogenicity [42]. In this regard, a VSV vector including portions of the VSV G and expressing HIV genes was found to be insufficiently attenuated for clinical evaluation when assessed for neurovirulence in nonhuman primates [43]. These investigators subsequently showed that safety and immunogenicity can be improved by genetic manipulation of the VSV genome but it remained unclear whether neurovirulence was associated with the VSV G or other genome manipulations [44]. Nevertheless, our ZEBOV vaccine is a G-deficient VSV vector [24] and thus lacks G-associated pathogenicity [41] as well as the target for VSV-specific neutralizing antibodies [45]. Aside from G, the VSV matrix (M) protein has been associated with cytopathic effects *in vitro* including the inhibition of host gene expression, induction of cell rounding and induction of apoptosis [46],[47]. It is largely unclear to what extent M alone contributes to pathogenicity, but inoculation studies with the VSV-based vaccines in different animal species (as described above) do not suggest a major pathogenic effect of the M protein *in vivo* [7],[13],[32].

Currently, the mechanism by which any filovirus vaccine confers protection in nonhuman primates is not well understood. Nearly all studies have detected modest to good humoral immune responses. For the VSVΔG/ZEBOVGP vaccine a humoral response is detected in macaques by day 14 after vaccination ([7]; TW Geisbert, unpublished observations). However, in the current study and consistent with an impaired immune system, our SHIV-infected macaques did not develop a humoral immune response by the time of ZEBOV challenge. Three animals developed modest anti-ZEBOV IgG titers 14 to 28 days after ZEBOV challenge. We are uncertain as to why four of the six VSVΔG/ZEBOVGP-vaccinated macaques survived ZEBOV challenge. Regardless of any humoral immune response elicited in these animals it is unlikely that antibody alone confers protection. Specifically, passive antibody studies in nonhuman primates using a variety of anti-ZEBOV immune reagents including polyclonal equine immune globulin [25], a recombinant human monoclonal antibody [48], and convalescent monkey blood [49] have uniformly failed to provide protection and more importantly have failed to provide any beneficial effect.

A number of studies have evaluated the cellular immune response in nonhuman primates vaccinated against EBOV and the results have been mixed with some studies showing a modest cellular response and other studies showing weak and/or no cellular immune responses [7],[9],[10]. However, it is likely that the intracellular cytokine assays that have been employed in some of these studies are not sensitive or thorough enough to detect a cellular immune response against ZEBOV. Indeed, it has been reported that the inability to demonstrate a robust cellular response may illustrate the limitation of the evaluation of cellular immune responses using small numbers of functional measurements (such as interferon-gamma) [50]. One interesting finding in the current study may begin to shed some light on the mechanism of protection elicited by the VSVΔG/ZEBOVGP. Notably, the two rhesus macaques that grouped together with the most severe loss of CD4+ T cells were the only animals that failed to survive ZEBOV challenge. This suggests that CD4+ T cells may play a role in mediating protective immunity in EBOV infections. CD4+ T cells have been shown to be depleted in nonhuman primate following ZEBOV infections [27],[51] and *in vitro* ZEBOV infection of human peripheral blood mononuclear cells causes massive bystander death of CD4+ T cells by apoptosis [52]. While rodents do not appear to faithfully reproduce ZEBOV infection of humans and nonhuman primates [53] studies have suggested that CD4+ T cells are required for protection of rodents against ZEBOV. Specifically, in a study using liposome-encapsulated ZEBOV antigens, Rao and colleagues showed that treatment of mice with anti-CD4 antibodies before or during vaccination abolished protection, while treatment with anti-CD8 antibodies had no effect, thus indicating a requirement for CD4+ T lymphocytes for successful immunization [54]. Similarly, depletion of CD8+ T cells did not compromise protection in mice indicating that CD8+ cytotoxic T cells are not a requirement for protection [32].

In conclusion, our results show that the VSV-based ZEBOV vaccine (VSVΔG/ZEBOVGP) did not cause any illness in immunocompromised SHIV-infected rhesus macaques and resulted in sufficient protective efficacy in all but the most severely compromised animals against a lethal ZEBOV challenge. Protection in the immunocompromised macaques appeared to be dependent on CD4+ T cells rather than the development of EBOV-specific antibodies. This provides strong support for the safety of the VSV-based vectors and further development of this promising vaccine platform for its use in humans. While these data are very encouraging, as the number of SHIV-infected macaques in the current study was small, additional safety studies will be needed in order to determine whether vaccines based on attenuated VSV will ultimately prove safe in immunocompromised humans.
